# Channel Allocation for Connected Vehicles in Internet of Things Services

**DOI:** 10.3390/s21113646

**Published:** 2021-05-24

**Authors:** Ahmed Abdulhakim Al-Absi, Mohammed Abdulhakim Al-Absi, Mangal Sain, Hoon Jae Lee

**Affiliations:** 1 Department of Smart Computing, Kyungdong University, 46 4-gil, Bongpo, Gosung, Gangwon-do 24764, Korea; absiahmed@kduniv.ac.kr; 2 Department of Ubiquitous IT, Graduate School, Dongseo University, 47 Jurye-ro, Sasang-gu, Busan 47011, Korea; d0185123@kowon.dongseo.ac.kr; 3 Division of Information and Communication Engineering, Dongseo University, 47 Jurye-ro, Sasang-gu, Busan 47011, Korea

**Keywords:** Internet of Things, transport system, channel allocation, V2V, channel access, radio propagation

## Abstract

Based on the existing Internet of Vehicles communication protocol and multi-channel allocation strategy, this paper studies the key issues with vehicle communication. First, the traffic volume is relatively large which depends on the environment (city, highway, and rural). When many vehicles need to communicate, the communication is prone to collision. Secondly, because the traditional multi-channel allocation method divides the time into control time slots and transmission time slots when there are few vehicles, it will cause waste of channels, also when there are more vehicles, the channels will not be enough for more vehicles. However, to maximize the system throughput, the existing model Enhanced Non-Cooperative Cognitive division Multiple Access (ENCCMA) performs amazingly well by connected the Cognitive Radio with Frequency Division Multiple Access (FDMA) and Time Division Multiple Access (TDMA) for a multi-channel vehicular network.However, this model induces Medium Access Control (MAC) overhead and does not consider the performance evaluation in various environmental conditions.Therefore, this paper proposes a Distributed Medium Channel Allocation (DMCA) strategy, by dividing the control time slot into an appointmentand a safety period in the shared channel network. SIMITS simulator was used for experiment evaluation in terms of throughput, collision, and successful packet transmission. However, the outcome shows that our method significantly improved the channel utilizationand reduced the occurrence of communication overhead.

## 1. Introduction

**Figure 1:**
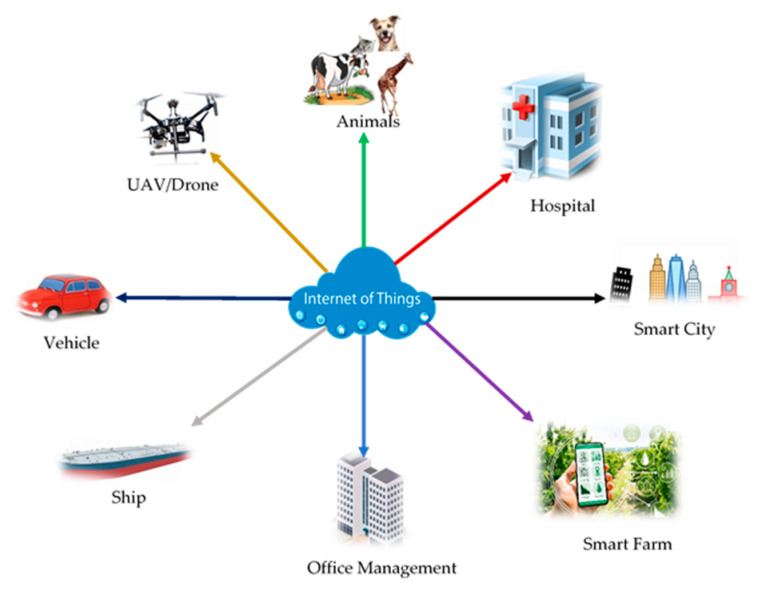
IoT application domains.

The growth trend of global Internet of Things applications is obvious, and it is currently in a period of strategic opportunities before the industrial explosion [1]. There are multiple assets in the consumer space that benefit from connectivity. Lights and air purifiers can be turned off according to the occupancy of the room. Window shutters can be automatically closed based on weather conditions and occasions. Energy and other resource consumption can be fully utilized based on usage patterns and forecasts. Due to the overlapping nature of application scenarios, different vertical fields have similar use cases. For Instanece, smart factory, logistics and transportation, public equipment, oil and gas, insurance, agriculturem, health service, Environmental monitoring, smart city, smat building, drones, andconnected car..ect (Figure 1). According to the latest McKinsey report, the global IoT market is expected to reach USD 11.1 trillion in 2025 [2], which means that the Internet of Things will likely represent more than 11% of the global economy and the future IoT market has unlimited potential. The rise of an industry is not only the demand of the market but also the synchronization of capital accumulation and technology. The development of the Internet of Things in Korea in the future is immeasurable, but it is currently limited by technology and security issues and has not yet entered a stage of rapid development. It is undeniable that IoT development is the future trend, so in the IoT industry, what opportunities can we grasp? What are the common IoT mobile application development types? The smart home is no stranger to everyone. It is mainly to connect the product and the user through the app, which can realize the control, appointment, linkage, and other functions of the smart device. At present, the more popular products of the smart home include smart door locks, smart curtains, smart lighting, smart security, smart home appliances, and other solutions and supporting products. Shared bicycles and car services belong to the Internet of Vehicles [3], with supporting products such as shared bicycle Bluetooth locks, car tire pressure monitoring, and smart parking. Through wearable devices, the perception of human body signs, statistics of users’ heart rate, exercise steps, and other information, combined with the function of big data analysis, to make intelligent and accurate judgments and services for users, will undoubtedly become a fast-paced immediate need. Environmental monitoring, as people’s living standards are getting higher and higher, people are paying more and more attention to health and environmental protection. We can combine Bluetooth to make an app that monitors the environment’s Pollution Monitoring (PM) value, temperature, humidity, etc. in real-time [4]. However, if it exceeds the limit, it will send an alarm message to the app and prompt the user to prepare for prevention. It is also a very marketable product. Industrial buildings, with the rapid development of society, urban management has become increasingly important. If you can use the Internet of Things, how much labor and financial costs will this save! For example, sensors can be installed on buildings to maintain and repair buildings and monitor safety by sensing temperature and humidity, which is also very helpful for building safety [5]. Connect everything and apply technical architecture as the cornerstone, the Internet of Things will be the next outlet of the Internet, and the related application technology architecture cannot be ignored. The realization of products requires technology as the cornerstone. Common application technology architectures are mainly divided into the following three types. Two-party communication architecture, the mobile app communicates with the smart device directly. This two-party communication architecture requires a custom communication protocol between the mobile app and the smart device. The data of the smart device is directly reported to the client, and then the client’s control instructions for the device are directly sent to the smart device [6]. The current communication protocol supports two methods based on Bluetooth and Socket under Wi-Fi. Three-party communication architecture, smart devices, business servers, and clients. This three-party communication architecture needs to implement a custom communication protocol between the smart device and the business server [7]. A stable connection channel is established between the smart device and the business server through Socket, and data reporting and command control are realized through remote connections. The three-party communication framework also has Wi-Fi and GPRS mode and Bluetooth modes. Wi-Fi and General Packet Radio Service (GPRS) mode: When the clientsare controlling the smart device, it will send instructions to the business server via Hypertext Transfer Protocol (HTTP) or Socket protocol. After receiving the instruction, the server sends the instruction to the smart device, and the smart device receives the instruction and makes feedback, report the information to the business end through User Datagram Protocol (UDP) or Transmission Control Protocol (TCP), and the business end receives the feedback data and sends it to the client for display.

**Figure 2:**
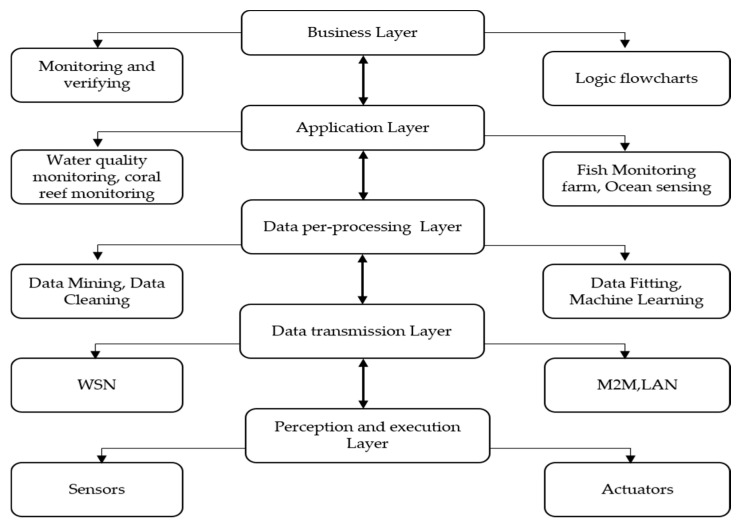
IoT Layer Architecture.

Bluetooth mode: The smart device establishes a connection channel with the client through the Bluetooth or Beacon protocol. The smart device reports data to the client through the connection channel. The client submits the data to the business server through HTTP or socket, and the business server performs analysis and processing, sends the data to the client for display, and the user can send instructions to the smart device through the data display on the client to control the device. Figure 2 shows the IoT layer architecture.

As concerns about the environment and sustainability are becoming more and more important to potential customers, the automotive industry is more inclined to electric vehicles than ever before. Today, automakers are also integrating connectivity and autonomous driving components in their vehicles to reduce travel time and improve the safety of drivers, passengers, vehicles, and the entire transportation system. However, from the perspective of the technological development path, smart cars are divided into three developments connected vehicles (CVs) [8], autonomous vehicles (AVs) [9], and electric vehicles (EVs) [10]. However, in this article, we are focus on connected vehicles. With the continuous increase in the number of connected vehicles, the traditional vehicular ad hoc networks (VANETs) are developed with the development of the Internet of Vehicles (IoV). In the autonomous electric vehicles environment [11], a new emerging application is found for enhancing traffic safety which can be categorized as a real-time system. Existing vehicle-to-vehicle safety systems and the new cooperative systems together withthe use of wireless data communication between vehicles helps in decreasing the number of accidents on the roads in the world i.e., before deadlines the messages are transmitted [12]. Moreover, in a wireless communication system, the requirements for high accuracy and low delay are imposed. For instance, lane departure warning messages combine assistance with emergency vehicle routing. In a traffic safety system, even after the delivery of information is correct, but later the deadline in the real-time communication system, not only unusable but can also have major consequences. This problem is pointed out in [13,14]. In most cases, the need for dedicated network architectures directly supports V2V communication when very low delays are required by traffic safety applications. For V2V ad-hoc communication in high-speed vehicular network environments, the IEEE 802.11p standard is meant, which states amongst other things that numerous data/packet exchanges within 50 milliseconds of time frame must be completed.

The original IEEE 802.11, meant for WLAN [15], contains two drawbacks within its medium access control (MAC) technique and carrier-sense multiple access (CSMA); which may cause unbounded delays before channel access and collisions on the channel. On the shared communication channel, the MAC protocol determines who has the correct data/packet to send next. In CSMA, the node will first know if a channel is free for a particular period, then the node will pass data/packets directly with the suggestion that the next node could have performed the same action, as an outcome in a collision on the channel. So due to the risk of the channel becoming occupied a node can experience very long channel delays. The whole IEEE 802.11 family uses CSMA, and it is a wired counterpart IEEE 802.3 Ethernet. Because of its direct implementation of the standard that results in affordable equipment, both WLAN and ethernet are successful. Because of this, WLANs and the ethernet are frequently applied to other domains than they are designed for. Even though CSMA is unsuitable because of the unbounded channel access delays for real-time V2V communication, ethernet has performed its way communication scene as there are many real-time systems are found [16]. By introducing more network equipment, the problems with the MAC protocol can be solved here, like switches and routers, and thereby the number of competing nodes on the shared channel is reduced, i.e., breaking down collision domains. But there is no such easy solution in the wireless domain because the wireless channel must be shared by all users. An interferer can easily confuse a geographical area when applying the CSMA algorithm in the wireless domain, although there is no real-time traffic, and the nodes in that area will delay their access. Since no access will occur if an activity is detected on the channel a wireless CSMA system is thus more susceptible to interference. The IEEE 802.11p is also called a dedicated short-range communication (DSRC) and is meant for VANETs [17]. Currently, DSRC is the only standard that supports direct V2V communication [18]. The original DSRC standard was a more application-specific standard, containing the entire protocol stack with (PHY) layers, MAC, and application layers found in Europe, Japan, and Korea. DSRC is used for hotspot communications such as electronic toll collection systems. PHY and its functions in 802.11p have been used in several articles [19,20,21]. System reliability (error probability) is mainly affected by PHY; However, if we cannot access the channel, we cannot take advantage of PHY. Although VANET cannot support deadlines in real-time, VANET uses CSMA as its MAC method. There are arguments that the CSMA problem is more evident under higher network load, and a traffic smoothing function that keeps the data traffic at an acceptable level can be introduced. In the centralized control network for restricted geographic areas, traffic facilitation [22] has been used. Due to its high dynamic characteristics and low latency requirements, VANETis not a geographically restricted area and cannot be predictable by a central controller.

The average delay can be reduced by traffic smoothing, but the main problem is with an unbounded worst-case delay which remains. While using CSMA [23], the issue with potentially access delays of an unbounded channel could be the use of self-organized time-division multiple access (STDMA), which is a decentralized, and yet predictable, MAC protocol with a limited channel access delay, making it fit for real-time ad-hoc vehicular networks [24]. In the system called automatic identification system, the STDMA algorithm is already in commercial use where it focuses on collision avoidance between vehicles [25].

In a VANET it is very important to reach the channel in a predictable time where the IEEE 802.11p medium access cannot guarantee that the channel will be accessed before the limited time has elapsed [26,27,28]. However, extensive investigations research has been conducted and the output showed that new MAC diagrams need to be designed for radio propagation in different environmental conditions, to maximize system throughput, reduce collisions, and use bandwidth more efficiently [29,30]. MAC designs future should consider these specifications when designing an effective MAC for VANET. 

The contributions of this research work are as follows:

- This work proposes an ideal access mechanism that considers the optimum target functionality of all traffic.
- This work presents two algorithms with distributed MAC for channel allocation.
- This model maximizes the throughput and reduces the overhead successfully compared with the existed model.
- The proposed model considered varied environments such as city, highway, and rural areas.

The paper is organized as follows: The literature review is discussed in Section 2. The proposed distribution medium for channel allocation is described in Section 3. Experimental results are presented in the penultimate section. At the last, the conclusion and future work is presented in the last section.

## 2. Literature Review

A VANET is a wireless mobile communication network [31]. However, in this paper we have introduced work in VANET channel allocation in conditions of high density and mobility under different environments. In [32] an adaptive multichannel MAC protocol called dynamic interval division multi-channel MAC has been proposed. Enables full use and adaptation of CCH and SCH duration depending on the real-time traffic load. According to the different types of frameworks, DID-MMAC divided the CCHI into three phases, SAP, BP, and PRP. Moreover, they developed a distributed algorithm that can calculate and determine the duration of the PRP communication. The authors in [33] examined the problem of throughput maximization of multiple access units and multi-channel opportunistic spectrum access networks. To solve an efficient solution design problem in an unknown and dynamic environment, the authors characterize the optimization issue as a cooperative game, justifying that this is a potential structured game. To solve the issue of scarcity spectrum, in [34] the authors divided the cognitive radio technology VANET (CR-VANET) into two stages: HCR-VANET and LCR-VANET and design the corresponding system problem model according to different scenarios to maximize the throughput. Because the communication capacity of the main vehicle (Leader) is limited, the authors in [35] propose a distributed network where each vehicle determines its speed only by contacting a short-distance vehicle. To improve VANET reliability and delay, a V2V resource allocation method based on C-V2X technology, the authors idea in [36] is that V2V communication between vehicles based on V2X cellular technology eliminates competitive latency and facilitates long-distance communication where the problem with optimizing resource allocation for cellular eNodeBs is choosing the best receiver for V2V correlation identification and appropriate channel assignment to reduce overall latency. In [37], the authors’ objectives are to get the throughput to the maximum of the whole system and solve the channel allocation issue in multi-channel cognitive vehicular networks. They suggested three effective algorithms to show that the issue is a non-linear integer programming NP-hard problem. In [38], the main problem with cellular V2V resource allocation is how to appropriately allocate V2V user spectrum resources and broadcast opportunities to improve network performance without causing significant interference with cell phone users [39]. The model [40] allows mobile phone users to specify how to operate the cellular or V2V link to establish the transmission [41]. MAC Protocol (IEEE 802.11p) is a combination of CSMA/CAwhich is widely used to access the network or the internet [42]. The enhanced distributed channel access is an update of the IEEE 802.11a to improve the system performance and provide QoS [43]. Moreover, the same connection is required for all applications of an intelligent transport system. Therefore, applications of traffic safety required connecting with real-time which most data packages must be delivered successfully within a certain period [26,27,28,44].Routing protocols are needed for V2V communications. At the MAC layer, a routing metric combines retransmission counts and hop counts. MAC layer is recommended with consideration of delay reduction and link quality. A cross-layer R-AOMDV routing protocol that is based on the new transmitting metric, is considered to make use of benefits of multi-path routing protocol, such as reduction of route detection frequency. In [45], a new logical model is constructed to derive MAC and application-level reliability metrics of IEEE 802.11 established one-dimensional (1-D) VANETs in highways, which include packet reception ratio, packet reception probability, awareness probability, and t-window reliability. The evaluation of point-to-point reception possibility the metrics derivation starts. The impact of concurrent transmissions and terminal problem coverage area is computed. The recommended facilities effect analysis of distance application-level analysis and DSRC fading channel. The systematic model takes non-saturated message arrival interval, the model takes IEEE 802.11 MAC, 1-D highway geometry, and Nakagami fading channel. The recommended model is verified/validated by extensive simulations.In [46], the doppler spectrum in wideband V2V communication channels. The authors examine the influence of mobile and stationary scattering clusters. In channel in which they simulate the model is an urban canyon oncoming environment. The author’s developed equal-delay contours in the context of geometry-based stochastic channel models (GSCMs), for accurate modeling of the location of both stationary and moving, scatters in vehicular environments. Then, they use the developed model to simulate the taps’ doppler spectra. numeric results prove that the doppler power spectra produced using the presented model are close to the outcomes took from measurements of V2V channels in an urban oncoming environment.Distributed rate control algorithm (DRCV) [47] is a congestion control algorithm exactly considered for VANETs. To make sure the channel is free for safety-critical, messages are given different priority levels. The rate of the less significant messages, such as decreases as the used bandwidth increases position update. To increase the packet delivery ratio by 15%, however, the author compared DRCV against no method of congestion control. Several technologies have been developed with CR [48,49,50] which is involved in exploration spectrum [51,52,53] and access of dynamic spectrum [54,55,56]. However, in the process of searching for solutions that provide better performance than the traditional proposals, these approaches are applied to the design of MAC mechanisms. In [57], the authors proposed a MAC protocol (enhanced non-cooperative cognitive division multiple access or ENCCMA) that utilizes cognitive radio technology. To maximize the system throughput, they connected the cognitive radio (CR) with FDMA and TDMA and designed MAC for a multi-channel vehicular network. Compared to the latest models, ENCCMA performs amazingly well. However, this model induces MAC overhead and does not consider the performance evaluation in various environmental conditions. However, the performance of the proposed model is evaluated in terms of throughput, collision, and successful transmission. The overall result shows that our method significantly improved the channel throughput and reduce the communication overhead.

## 3. Distributed Medium for Channel Allocation

A VANET contains the characteristics of wireless ad-hoc networks, which make communication between vehicles, and communication between vehicles and roadside infrastructure. Channel allocation problems and channel access problems are the main differences between different MAC mechanisms. The channel allocation problem is mainly responsible for allocating corresponding communication channels for different vehicle nodes and eliminating the conflicts between communication links caused by channel switching; the access control problem is mainly responsible for solving the timing conflicts when different vehicle nodes access the channel. In the traditional single-channel MAC protocol, all nodes share a channel for transmission. In the vehicle environment, due to many vehicle nodes and roadside infrastructure, the single-channel allocation mode will greatly limit the network throughput. Therefore, the multi-channel allocation method is adopted on the Internet of Vehicles. Nodes can use multiple channels, and vehicles can work on different channels for data transmission, which can improve network throughput. 

Here, we propose an uncomplicated, efficient, unshared, and shared channel for vehicular networks. This model aims to maximize system throughput and reduce MAC collisions (overhead). The list of notations and symbols used in this paper are given in Table 1.

- Un-Shared Channel

Assume that $T_{x}$ is the set of channels for vehicle x. This technology frequently dedicates channels to vehicles to maximize network throughput. On each channel assignment iteration, each vehicle x computes the throughput gain if the optimum channel is set with the following condition:

(1) $$y_{x}^{\prime }=\underset{y\in T_{z}}{argmax}l_{x,y},$$

This productivity gain can be calculated as:

(2) $$\delta C_{x}=SC_{x}^{z}-C_{x}^{q}=\left[1-\left(1-l_{xy_{x}^{\prime }}\right)\prod_{y\in T_{x}}\left(1-l_{xy}\right)\right]-\left[1-\prod_{y\in T_{x}}\left(1-l_{xy}\right)\right]=l_{xy_{x}^{\prime }}\prod_{y\in T_{x}}\left(1-l_{xy}\right)$$

**Algorithm 1 Unshared Channel**
Step 1. Input set of accessible channel $T_{z}=\left\{1,2,3,\ldots ,T\right\}$ & $T_{x}=\emptyset$ for x=1,2,3,…,R vehicles Step 2. For x=1;x<=R;R++ Step 3. $y_{x}^{\prime }=\underset{y\in T_{z}}{\mathrm{argmax}}l_{x,y}$. Step 4. If ($T_{x}=\emptyset$) then Step 5. Obtain ${\delta C}_{x}=C_{x}^{z}-C_{x}^{q}$, where $C_{x}^{q}$ and $C_{x}^{z}$ are the throughputs before and after channel allocation $y_{x}^{\prime }$. Step 6. Else Step 7. Obtain ${\delta C}_{x}=l_{{\mathrm{xy}}_{x}^{\prime }}$. Step 8. End If Step 9. End For Step 10. $x^{\prime }=\underset{x}{\mathrm{argmax}}{\delta C}_{x}$. Step 11. Allocate channel $y_{x^{\prime }}^{\prime }$ to vehicle $x^{\prime }$. Step 12. Update $T_{z}=T_{z}/y_{x^{\prime }}^{\prime }$. Step 13. If $T_{z}$ is empty, terminate the process. Step 14. Else, go to step 2.

It can be seen in the Equation (2) that $T_{x}$, $\prod_{y\in T_{x}}\left(1-l_{xy}\right)$ tends to zero if the $T_{x}$ increases. $\delta C_{x}$ will decrease with each iteration of the allocation.

- Shared Channel

The channel allocation model consists of two steps. First, to calculate the channel allocation information for a single vehicle. Second, it then deals with multi-user channel allocation by assigning channels assigned to specific vehicles to other vehicles.

Determines the optimum channel available to the user based on productivity gain requirements. Users do not share channels here; users enter the channel during a specified period and leave the channel so that other users can access it. However, this bandwidth cannot be used efficiently. This is because the channels are not shared. To solve this problem, we present a novel MAC distribution for VANET environments as shown:

(3) $$\delta C_{x}^{T,b}\left(y\right)=\left(1-\frac{1}{T}\right)\left(1-D\right)l_{xy}\left(\prod_{o\in T_{x}}{\bar{l}}_{xo}\right)*\left(1-\prod_{o\in T_{*}^{S}}{\bar{l}}_{xo}\right)\sum_{n=1}^{T}\left[{\bar{l}}_{x_{n}y}\left(\prod_{m=1,m\neq n}^{T}l_{x_{m}y}\right)\right]$$

**Algorithm 2 Shared Channel**
Step 1. Input set of assigned channels ∀ vehicles $T_{x}=\emptyset$ for x=1,2,3,…,R and $D_{o}$ Step 2. Execute Algorithm 1 to get channel allocated for a single vehicle. Step 3. Let the set of channels that are shared by j vehicles be $P_{j}$ and $F_{y}$ be the group of vehicles which share channel y and set $F_{y}^{T}=F_{y}\;\forall y=1,2,3,\ldots ,T$. Step 4. Process=1; o=1; UpdateOvd=0. Step 5. While Process=1 do Step 6. Obtain the set of channels $P_{o}$ shared by o vehicles Step 7.  For $y=1;y\leq \left|P_{o}\right|;y++$ Step 8.   For j=1;j≤R;J++ Step 9.    If $j\in F_{y}$ then Step 10.     ${\delta C}_{j}^{o,b}\left(y\right)=0$. Step 11.   Else Step 12.     User j computes ${\delta C}_{j}^{o,b}\left(y\right)$ considering that channel y is assigned to vehicle j. Step 13.   End If Step 14.    End For Step 15.   $j_{y}^{\prime }=\arg\underset{j}{\max}{\delta C}_{j}^{o,b}\left(y\right)$. Step 16.  End For Step 17. $j_{y}^{\prime }=\arg\underset{y}{\max}{\delta C}_{j_{y}^{\prime }}^{o,b}\left(y\right)$. Step 18. If ${\delta C}_{j^{\prime }}^{o,b}\left(y_{j^{\prime }}^{\prime }\right)\leq \epsilon$ and UpdateOvd=1 then Step 19.  Set Process=0. Step 20.  Go to step 35. Step 21. End If Step 22. If ${\delta C}_{j^{\prime }}^{o,b}\left(y_{j^{\prime }}^{\prime }\right)>\epsilon$ then Step 23.  Provisionally allocate channel $y_{j^{\prime }}^{\prime }$ to vehicle $j^{\prime }$, i.e., update $F_{y_{j^{\prime }}^{\prime }}^{T}=F_{y_{j^{\prime }}^{\prime }}\cup \left\{j^{\prime }\right\}$. Step 24.  Compute A and D. Step 25.  If $\left|D-D_{o}\right|>\epsilon_{D}$ then Step 26.   Set:Process=1. Step 27.    Return to Step 7 using the updated $D_{o}=D$. Step 28.  Else Step 29.   Update $F_{y_{j^{\prime }}^{\prime }}=F_{y_{j^{\prime }}^{\prime }}^{T}$ (i.e., allocate channel $y_{j^{\prime }}^{\prime }$ to vehicle $j^{\prime }$), compute A&$D_{o}$ with $F_{y_{j^{\prime }}^{\prime }}$, & update $P_{o}$. Step 30.   Update UpdateOvd=0. Step 31.  End If. Step 32. End If. Step 33. Return to step 7. Step 34.  o=o+1. Step 35. End While

Usually, one does not need many channels to achieve maximum throughput. Assuming each vehicle has spectrum access with a probability of 0.8, and the return earned by a qualified vehicle with three dedicated channels is greater than $1-{\left(1-0.8\right)}^{3}=0.992$, which is rated at a maximum throughput of less than 1%. We can calculate the set of channels assigned to each vehicle using Equation (3) to calculate the throughput.

- Contention window computation

Contention window A is computed to reduce the probability of a collision between vehicles. There is a trade-off between MAC protocol overhead and collision potential, and it is affected by A. That is, the smaller the value of A, the higher the collision probability, but less than the MAC load, vice versa. Each vehicle chooses some equal time to pull back. As a result, the probability of a first collision increases as the number of vehicles involved decreases the higher the probability of a collision.

Let $L_{u}$ be the probability of the first collision. Consider the constraint $L_{u}\leq \epsilon L$, where ϵL defines the trade-off between management overheads and collision probability to locate the contention window A. Given the presence of r vehicles in the contention stage, we evaluate $L_{u}$ as a function of A. Without losing generality, let us consider the back-off time of r vehicles as $g_{1}\leq g_{2}\leq g_{3}\leq \ldots \leq g_{r}$. Assuming, r vehicle is present in the contention stage, the conditional probability of the 1st collision can be indicated as:

(4) Lu(r)=∑y=2rL(yvehiclescollide)=∑y=2r∑x=0A−2Ury(1A)y(A−x−1A)r−y

As each term in double addition indicates the collision probability of y collision when determining back-off value concerning x. However, the probability of the first collision can be calculated as:

(5) $$L_{u}=\overset{R}{\underset{r=2}{\sum }}L_{u}^{\left(r\right)}*L\left\{rvehiclecontend\right\}$$

where L{rvehiclecontend} is the probability that r vehicles will participate in the contention stage, and $L_{u}^{\left(r\right)}$ is calculated using Equation (4). To evaluate $L_{u}$, we conclude L{rvehiclecontend}. 

We suggest a distributed medium access control that divides time into an identical time of length δs. The total amount of time the user stays in xth RSU range is obtained as:

(6) $$N_{x}=\lfloor \frac{2K_{x}}{l\delta s}\rfloor$$

The Nth time slot when the user in a range of $g^{th}\;RSU$ is obtained as follows:

(7) $$B\left(x,N\right)=\overset{x-1}{\underset{h=0}{\sum }}N_{x}+N,\;\;\forall N\in \left\{1,\ldots ,\;N_{x}\right\}$$

where $N_{0}$ = 0. The set of time slots in xth RSU for timeline representation is $N_{x}=\left\{B\left(x,\;1\right),\;\ldots ,\;B\left(x,\;M_{x}\right)\right\}$. The communication optimal problem of users considered as a finite-horizon sequential quality specifies a problem. The time/iteration of the user is:

(8) $$n=\mathbb{N}=\underset{x\in X}{\cup }N_{x}=\underset{x\in X}{\cup }\;\{B\left(x,1\right),\ldots .,\;B\left(x,N_{x}\right)\}$$

where ℕ represents the set of all slots within the xth range, and the method m∈M=[0, M] represents the effective size of the transmitted data packet. If we represent the number of users in xth RSU coverage range as $v\in V_{x}=\left\{1,\ldots ,\;V_{↑},\;x\right\}$ then $g^{+ve}\in G_{x}=\left\{z_{x}\left(v\right):v\in V_{x}\right\}$.

The user has two possible states at any modes $\left(m,\;g^{+ve}\right)$, that can be represented as:

(9) a∈A={0,1}

where states a=1 indicates the user has agreed to send the request, and a=0 indicates that the user does not approve the sent request.

The cost incurred at mode $\left(m,\;g^{+ve}\right)$ with instance a in the time slot $n\in N_{x}$ in the xth coverage area is:

(10) $$b_{n}\left(m,\;g^{+ve},a\right)=az_{x}\;\forall n\in N_{x}$$

For example, when the user leaves the xth coverage area $B\left(X,\;N_{x}+1\right)$, the overhead occurs for the user because the transmission packet is not complete. The packet transmission can be computed as:

(11) $${\hat{b}}_{B\left(X,N_{x}+1\right)}\left(m,g^{+ve}\right)=r\left(m\right),$$

where r(m)≥0 is the non-abbreviated parameter of m with r(0)≥0, which is related to the QoS of the application. Thus, the transmission costs resulting from the subscriber include two things.First, the cost of communication for each time slot in Equation (8). Second, in Equation (9) the overhead occurs after being out of the xth coverage range. The possibility of transitional mode $\left(\left(\bar{m},\;\bar{g^{+ve}}\right)|\left(m,\;g^{+ve}\right),\;a\right)$ is the probability that the network will be in mode $\left(\bar{m},\;\bar{g^{+ve}}\right)$ if sates a is obtained at mode $\left(m,\;g^{+ve}\right)$ at time slot n∈M. However, the transmission from $g^{+ve}$ to $\overset{=}{g^{+ve}}$ is not a specified by m but specified by time n, so we have:

(12) $$g_{n}\left(\left(\bar{m},\;\bar{g^{+ve}}\right)|\left(m,\;g^{+ve}\right),\;a\right)=g_{n}\left(\left(\bar{m}\right)|\left(m,g^{+ve}\right),a\right)g_{n}\left(\overset{=}{g^{+ve}}|\;g^{+ve}\right)$$

With state a=1, we obtain:

(13) $$g_{n}(\bar{m}|\left(m,\;g^{+ve}\right),\;1)=\left\{ \begin{array}{ll} g^{+ve}, & if\;\bar{m}={\left[m-d_{n}\delta n_{packet}\right]}^{*},\\ 1-g^{+ve}, & if\;\bar{m}=m,\\ 0, & Otherwise, \end{array}\right.$$

where ${\left[y\right]}^{+}=max\left\{0,y\right\}.$ The first and second cases show positive and negative data transfer, respectively. With states m=0, so we obtain:

(14) $$g_{n}\left(\bar{m}|\left(m,\;g^{+ve}\right),0\right)=\{\begin{array}{c} 0,\;\;\;\;if\;\overset{=}{m}\;\neq m,\\ 1,\;\;\;Otherwise, \end{array}$$

where the remaining packets are not resized to be transmitted. In later subsection the derivation of $g_{x}\left(\overset{=}{g^{+ve}}|\;g^{+ve}\right)$ is discussed.

Let $\Delta_{n}:\;M*G_{x}\to K$ be the optimal QoS connection for the subscriber under the specified mode $\left(m,g^{+ve}\right)$ at slot time $n\in N_{x}$ in the xth coverage area. The objective functionnow expresses as QoS as $\left(\Delta_{n}\left(m,g^{+ve}\right),\;\forall \;m\in M,g^{+ve}\in G_{x},n\in N_{x}\forall x\in X\right).$ We consider Da realistic set of D. The time slot n can be performed as $\left(m_{n}^{D},\;g_{n}^{+ve,D}\right)$ if D is used. The participantsaim at reducing the cost and satisfying objective function as a problem of improvement:

(15) $$\underset{D\in D}{min}Y_{D}\left(M,\;g_{1}^{+ve}\right)\left[\overset{X}{\underset{x=1}{\sum }}\left[\overset{N_{x}}{\underset{k=1}{\sum }}b_{B\left(x,N\right)\left(m_{B\;\left(x,N\right),}^{D}\;g_{g\left(x,N\right)}^{+ve,D},\;\Delta_{B\left(x,N\right)}\left(m_{B\left(x,N\right)}^{D},\;g_{B\left(x,N\right)}^{+ve,D}\right)\right)}\right]+{\hat{b}}_{B\left(x,N_{x}+1\right)}(m_{B\left(x,N_{x+1}\right),\;}^{D}\;g_{B\left(x,N_{x+1)\;}\right)}^{+ve,D}\right],$$

where $Y_{\left(D,\;g_{1}^{+ve}\right)}$ is the likelihood with honor to probability distribution by function D with mode $\left(M,\;g_{1}^{+ve}\right)$ at slottime n=B(1,1)=1.

Let us consider that there is only one RSU and the user arrival of α is considered and not known the traffic pattern. The transitionprobability of $g^{+ve}$ is obtained as:

(16) $$g_{n}\left(g^{+ve^{\prime }}|\;g^{+ve}\right)=g_{n}(g\;\left(\bar{v}\right)|g\left(v\right))=g_{n}\left(\bar{v}|v\right)=\;\{\begin{array}{c} \frac{{\left(\alpha \delta n\right)}^{\bar{v}-v+c_{n+1}}}{\left(\bar{v}-v+c_{n+1}\right)!\beta_{n}\left(v\right)},\;\;\;\;\;\;\;\;\;\;\;\;\;\;\;\;\;\;\;\;\;\;\;if\;v-c_{n+1}\leq \;\bar{v}\;\leq V_{↑}\;,\\ 0,\;\;\;\;\;\;\;\;\;\;\;\;\;\;\;\;\;\;\;\;\;\;\;\;\;\;\;\;\;\;\;\;\;\;\;\;\;\;\;\;\;\;\;\;\;\;\;\;\;\;\;\;\;Otherwise, \end{array}$$

where $\beta_{n}\left(v\right)=\sum_{i=0}^{V_{↑-v+c_{n+1}}}\frac{{\left(\alpha \delta n\right)}^{i}}{i!}$ is the function of normalization since $g^{+ve}$ is a reduction function of v, and there is one-to-one assignment among $g^{+ve}$ and v as shows in the first two in Equation (11) and the third equality depicts the probability with $\bar{v}-v+c_{n+1}$ arrival because of the Poisson process and $c_{n+1}$ displacement is inevitable for instance $n+1.\bar{v}$ is bordered by the upper $V_{↑}$ and lower limited by $v-c_{n+1}\geq 0$ when there is no subscriber arrival.

Given the problem of RSU={1} Equation (10) we can simplify it as follows:

(17) $$\underset{D\in D}{min}Y_{D,\left(M,\;g_{1}^{+ve}\right)}\left[\overset{N}{\underset{n=1}{\sum }}b_{n}\left(\left(m_{n}^{D},\;g^{+ve,D},\;\Delta_{n}\left(m_{n}^{D},\;g_{n}^{+ve,D}\right)\right)\right)+{\hat{k}}_{M+1}\left(m_{n+1}^{D},\;g_{n+1}^{+ve,D}\right)\right]$$

Let $l_{n}\left(m,\;g^{+ve}\right)$ be the minimum cost where the users sometimes must pay from time to time N+1 when the coverage range is in mode $\left(m,\;g^{+ve}\right)$ before deciding time slot n∈N. The optimization of minimum entire cost at various modes for n∈N is as follows:

(18) $$l_{n}\left(m,\;g^{+ve}\right)=\underset{a\in A}{min}\left\{\gamma_{n}\left(m,\;g^{+ve},\;a\right)\right\}$$

where:

(19) $$\gamma_{n}\left(m,\;g^{+ve},\;a\right)=b_{n}\left(m,\;g^{+ve},\;a\right)+\sum_{\bar{m}\epsilon M}\sum_{g^{+ve,\;\epsilon G}}g_{n}\left(\left(\bar{m},\;g^{+ve\prime }\right)|\;\left(\bar{m},\;g^{+ve}\right),\;o\right)l_{n+1}\left(\bar{m},\;g^{+ve\prime }\right)$$

(20) $$=ou+\underset{g^{+ve\prime \in G}}{\sum }g_{n}\left(g^{+ve^{\prime }}|\;g^{+ve}\right)\left[\begin{array}{c} og^{+ve}l_{n+1}\left({\left[\bar{m}-d_{n}\delta n_{packet}\right]}^{*},\;g^{+ve\prime }\right)\\ +\left(1-og^{+ve}\right)l_{n+1}\left(m,\;g^{+ve\prime }\right) \end{array}\right]$$

Equation (16) gives the actual and projected future cost of choosing o for the remaining time slots of the coverage area. Using Equations (10)–(12) and (17) computes directly Equation (16). For example, the interval n=N+1, we possess the limiting factor as follows:

(21) $$l_{n+1}\left(m,\;g^{+ve}\right)={\hat{b}}_{n+1}\left(m,\;g^{+ve}\right)=r\left(m\right)$$

However, for $\gamma_{n}\left(m,\;g^{+ve},\;a\right),\;\forall n\in N$, the value is calculated as follows:

(22) $$\begin{array}{c} \gamma_{n}\left(m,\;g^{+ve},\;a\right)=au+\overset{V_{↑}-c+n_{n+1}}{\underset{j=0}{\sum }}\frac{{\left(\alpha \delta n\right)}^{j}}{j!\beta_{n}\left(v\right)}\\ \;\;\;\;\;\;\;\;\;\;\;*\;[ag^{+ve}l_{n+1}\left(\left[m-d_{n}\delta n_{packet}\right],\;z\left(v+j-c_{n+1}\right)\right)\\ \;\;\;\;\;\;\;\;+\left(1-ag^{+ve}\right)l_{n+1}\left(m,\;z\left(v+j-c_{n+1}\right)\right)] \end{array}$$

where $v=z^{-1}\left(g^{+ve}\right)$ is the density of vehicles in the coverage area of RSU. Using Equation (11), the result followed immediately by computing Equation (15). However, the packet size m must be minimal to be sent at a lower cost $l_{n}\left(m,\;g^{+ve}\right)$ which can be confirmed if $l_{n}\left(m,\;g^{+ve}\right)$ the parameter is not less than/equal to the previous one in $m,\;\forall g^{+ve}\in G,\;n\in N.$ Therefore, the optimal target function $D^{*}$ is achieve as follows:

(23) $$D^{*}=\left(\Delta_{n}^{*}\left(m,\;g^{+ve}\right)\right),\;\forall m\in M,\;g^{+ve}\in G,\;n\in N,$$

where:

(24) $$\Delta_{n}^{*}\left(m,\;g^{+ve}\right)=\arg\underset{a\in A}{\min}\left\{\gamma_{n}\left(m,\;g^{+ve},\;a\right)\right\},$$

However, the solution of problem Equation (17) is the objective parameter $D^{*}$. In the next section an experimental study is carried out to evaluate the performance of the existing model over the proposed model.

## 4. Results

Experiments were conducted on a 64-bit I-5 processor with 32GB RAM, windows 10. The SIMITS simulator [57] tool is used for experimental evaluation. SIMITS is a software communication in the field of the intelligent transport system (IST), allow us to measure the performance of different MAC (RR-Aloha, Slotted-Aloha, and ENCCMA) and change the parameters (number of vehicles). The proposed model and the existing model are written in a C# object-oriented programming language using Visual Studio framework 4.5, 2012. In this experiment, vehicle speed varies at 20 per frame, and stationary vehicles of 30, 60, and 90. The performance of city, highway, and rural environments is calculated in both DMCA and ENCCMA. For simulating and modeling the C.H.R [58,59,60,61,62,63,64] environmental conditions, we considered the parameters presented in [65]. Table 2 is illustrating the evaluation simulation parameters. The simulation parameter being considered for evaluation is shown in Table 3.

### 4.1. Throughput, Data Transmission, and Collision Performance of the DMCA Model 

**Figure 3:**
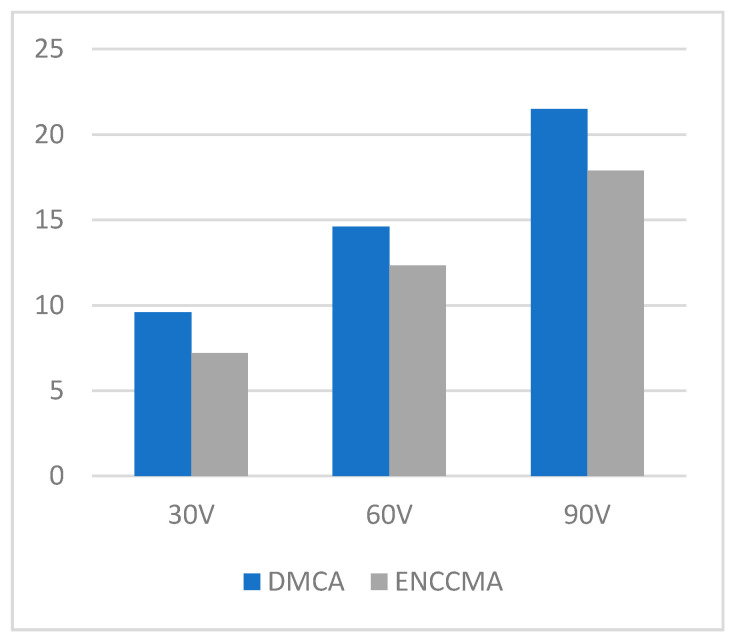
Throughput for the city environment.

**Figure 4:**
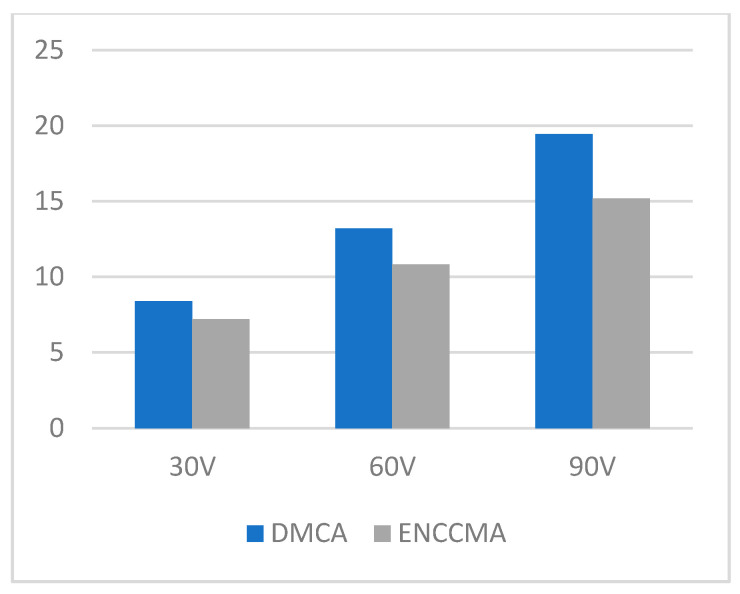
Throughput for the highway environment.

**Figure 5:**
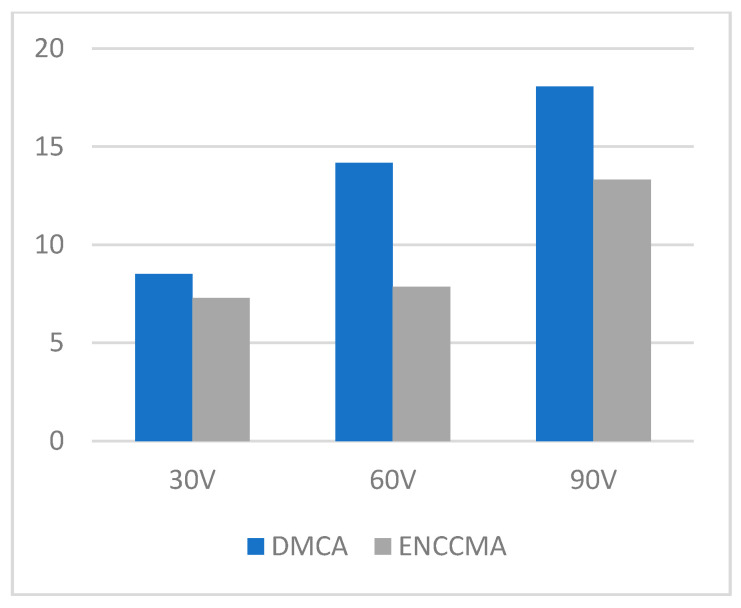
Throughput for the rural environment.

**Figure 6:**
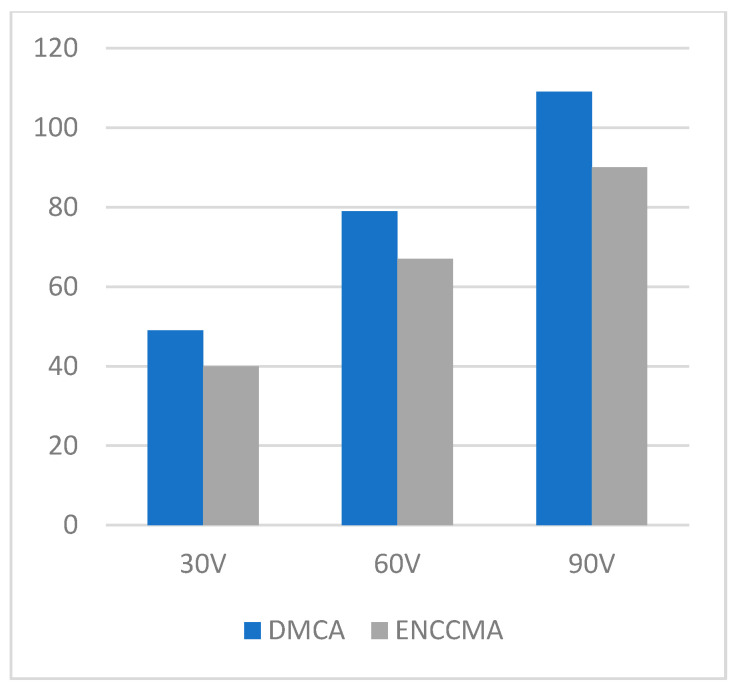
Data transmission for the city environment.

**Figure 7:**
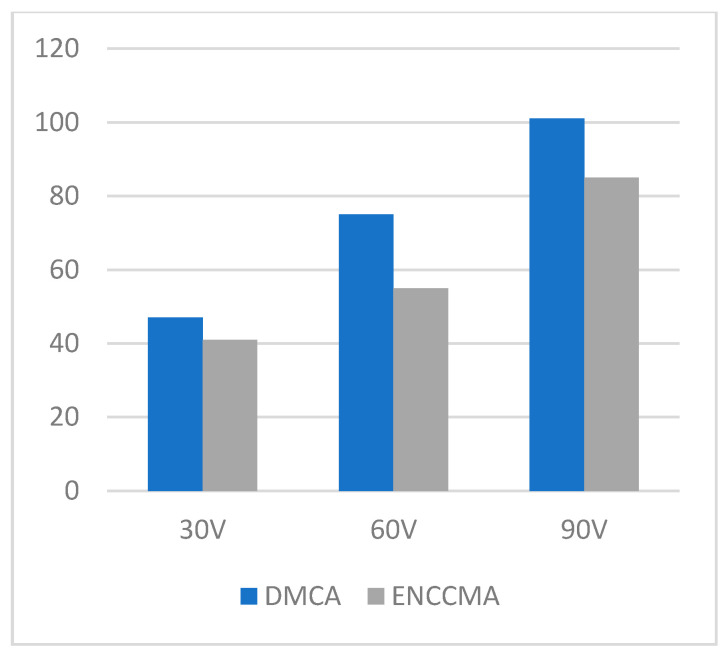
Data transmission for the highway environment.

An experiment is performed to check the performance of DMCA and ENCCMA, considering the processing speed of each channel in city, highway, and rural areas with different vehicle densities. In Figure 3, compared to ENCCMA, DMCA throughput increases by 17.17%, 10.25%, and 9.26% respectively. Compared to other environments, the average productivity of DMCA in the city environment increases by 12.23%. In Figure 4, compared to ENCCMA, DMCA productivity increases by 15.12%, 19.01%, and 22.93%, using 30, 60, 90 vehicles, respectively. Therefore, compared to ENCCMA, the average productivity of DMCA in highway environments increases by 19.026%. Figure 4 illustrates the processing performance for a rural environment. In Figure 5, compared to ENCCMA, DMCA increased productivity by 17.42%, 41.38%, and 17.17% of varied vehicles 30, 60 and 90, respectively. Compared to the rural environment, the average productivity of DMCA is 25.32% higher than that of the ENCCMA average. Figure 6, considering that there are 30, 60 and 90 vehicles respectively and compared to ENCCMA, DMCA increases the data transmission by 15.58%, 12.06%, and 7.54%, respectively. In Figure 7, compared to ENCCMA, DMCA studies 30, 60 and 90 vehicles and improve the data packets for highway environment by 15.89%, 29.00% and 22.90%, respectively. 

**Figure 8:**
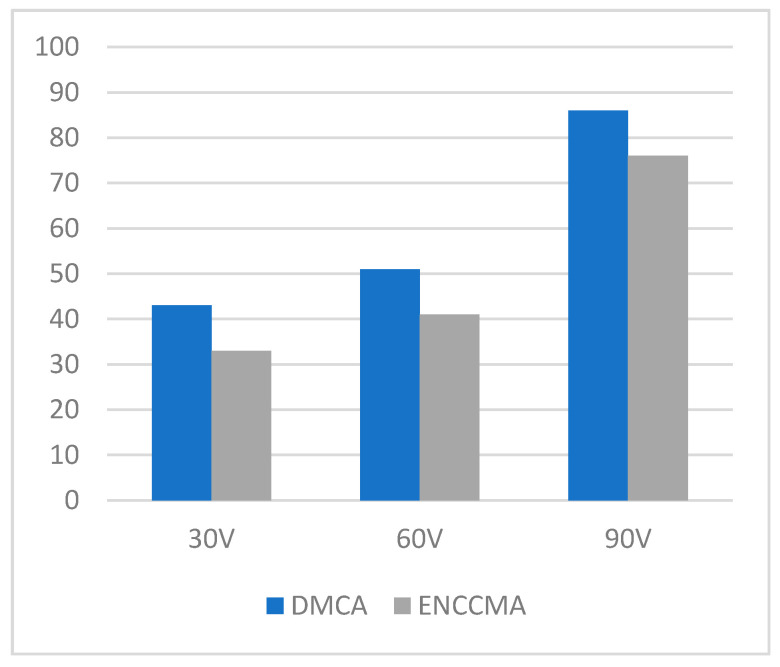
Data transmission for the rural environment.

**Figure 9:**
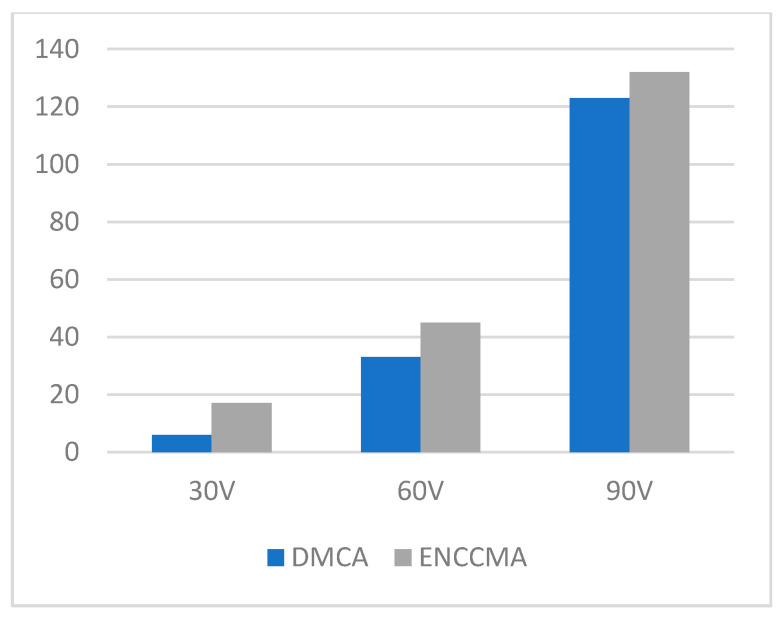
Data collision for the city environment.

**Figure 10:**
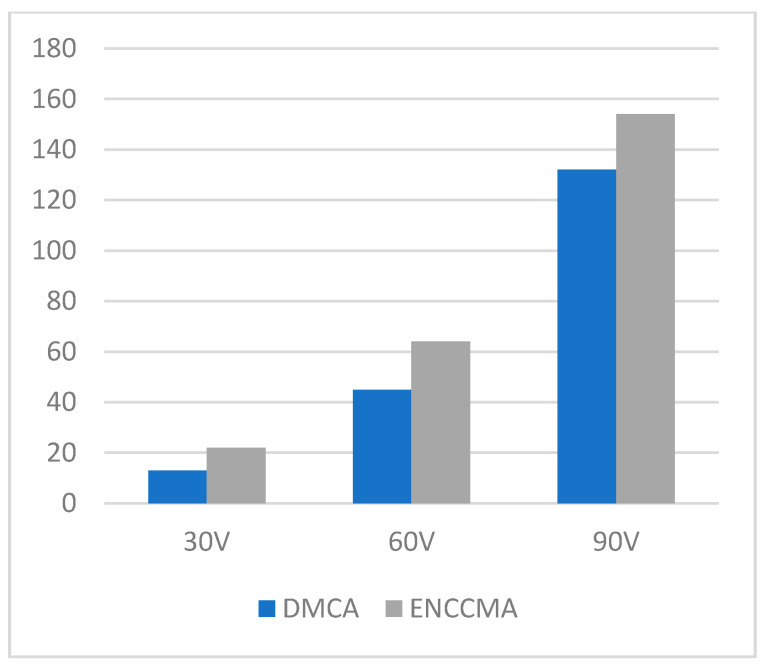
Data collision for highway environment.

**Figure 11:**
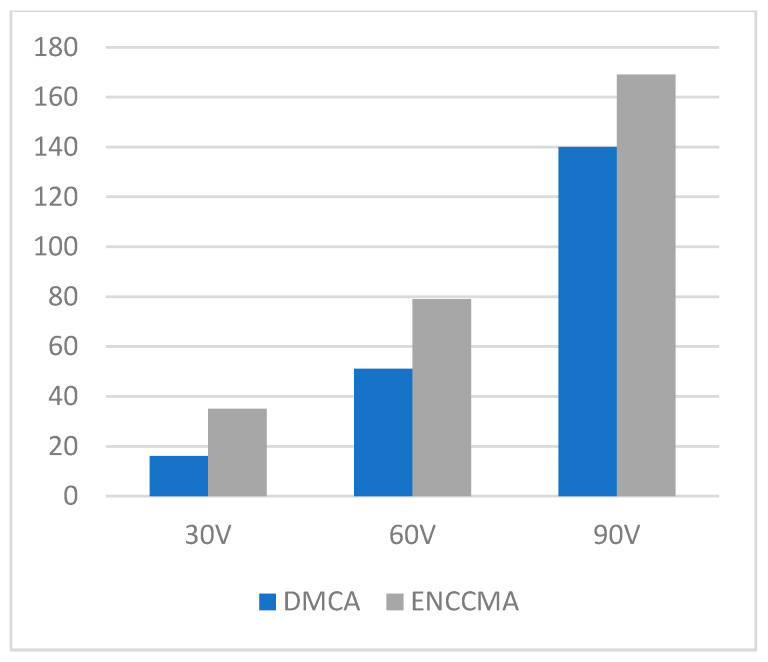
Data collision for the rural environment.

Figure 8 illustrates the performance of packet transmission in a rural environment. Looking at 30, 60 and 90 different vehicles in Figure 7, DMCA increases by 24.25%, 20.60%, and 12.62% compared to the ENCCMA. In Figure 9, DMCA provided 30, 60, and 90 vehicles, respectively, resulting in reductions of 65.70%, 27.66%, and 6.3% in data collisions for the city environment. As shown in Figure 10, given that there are 30, 60 and 90 vehicles and compared to ENCCMA, DMCA reduces collisions in the highway environment by 28.77%, 33.81%, and 16.58%, respectively. Figure 11 shows that DMCA reduces the collisions in the rural area by 52.51%, 34.76%, and 9.53% compared to the ENCCMA. The overall results show that DMCA performs well outperforming ENCCMA in different varied vehicles and different environmental conditions. This indicates that the proposed model is adaptable.

### 4.2. State of the Art Technology Comparison

Table 4 shows the comparison between DMCA with the state-of-the-art technology. To improve the system efficiency, DMCA supports the distribution channel sharing mechanism in V2V environments and helps the system to achieve maximum throughput and minimum overhead. The ENCCMA adopts the enhanced non-cooperative cognitive division multiple access (ENCCMA) [57] real-time MAC communication protocol. To provision real-time access, the ENCCMA combines time division multiple access (TDMA), frequency division multiple access (FDMA), and CR techniques. The ENCCMA medium access control protocol avoids signaling, this aids in enhancing the system’s efficiency. However, ENCCMA did not consider message authentication and security for personal user information. Reference [66] evaluated the performance of transmission of packet data considering different environments. However, they did not consider the movement and the numbers of the vehicles. In [67,68], the author performed an experimental analysis that considers different speeds for collision performance evaluation. However, performance evaluation under other environmental conditions is not considered. Our model considers different environmental conditions with varied density, and speeds considering throughput, collision, and transmission success performance. A comprehensive survey reveals the model’s effectiveness compared to the state-of-the-art technology. Table 5 shows the comparison of resource allocation techniques with our model for the V2X network. However, compared to other existing works, our model is the only scheme uses different environment and significantlymaximize the throughput and minimized the collision. In [69], the RA Scenario system was studied outside the scope in which the network infrastructure allocates resources to vehicles according to the vehicle’s estimated location. The performance of the resource allocation plan is analyzed for unexpected and planned services. In [70], the RA algorithm has been proposed to improve network connectivity. The authors of [70] assessed the performance of the proposed scheme using NS-3. This takes into account road of two-way, four-lane, and randomly 1 km vehicles distributed. The vehicle’s transportation radius is 50 meters, and the driving speed ranges from 20 to 60 km/h with the change of the number of vehicles. In [71], a simulation was performed considering the layout of a 20 m × 500 m road with the base station positioned in the center of the long side. Vehicles are randomly placed on the road and have a random speed from 0 to 100 km/h. In [72], the scheme proposed an optimization problem aimed at reducing computational complexity and maximizing the overall network percentage. The authers in [73] proposed a scheme for communiaton support of V2X in a D2D cellular system. Here, the existing cellular link strategy supported the V2I communication is by a traditional cellular uplink strategy aand V2V communication takes effect through the reuse of D2D communications [74]. An optimization problem was proposed to increase the overall navigation area of the V2I links in the vehicle while meeting the latency of the V2V links requirements.

## 5. Conclusions 

In the traditional single-channel MAC protocol, all nodes share a channel for transmission. In a vehicle-mounted environment, due to the many vehicle nodes and roadside facilities, the single-channel allocation mode will greatly limit the network throughput. Therefore, a multi-channel allocation method is adopted on the Internet of Vehicles. Each node can use multiple channels, and vehicles can work on different channels for data transmission, which can improve network throughput. However, due to the highspeed of vehicle movement, the connection status between the vehicles changes rapidly, the signal is sometimes missing, the communication effect is not ideal, and the channel allocation is particularly difficult. Therefore, a more reasonable channel allocation strategy is needed for this high-speed mobile environment or even Some special places. 

The existing proposed MAC protocol ENCCMA utilizes CR technology. The CR connected with FDMA and TDMA to design MAC for multi-channel vehicular networks. Compared to the latest models, ENCCMA performs amazingly well. However, performance evaluation is not considered in various environmental conditions. Extensive research has been conducted that a new MAC needs to be designed for radio propagation in different environmental conditions, to maximize system throughput, reduce collisions, and use bandwidth more efficiently. However, MAC designs future should consider these specifications when designing an effective MAC for VANETs.

This paper proposes a distributed channel allocation strategy, by dividing the control time slot into an appointment period and a safety period. In the appointment period, the time is divided into multiple time slices and allocated to each vehicle. The vehicle belongs to itself. Channel reservations are made within the time slice, which can reduce the occurrence of reservation information collisions. Then when the vehicles are dense and the transmission time slot is not enough for vehicle transmission, the reservation can be made without affecting other vehicle reservations and safety message transmission. Data transmission is carried out regularly, thereby improving channel utilization. By implementing the protocol proposed in this paper in the SIMIT simulator, the outcome shows that our method improved the channel utilization throughput and reduce the collisions. Experiments were performed to evaluate performance in terms of channel utilization, overhead, and successful data transmission, given in a high vehicle congested network. Experiments have shown that the proposed MAC design is adaptable to various environments (city, highway, and rural).

As there are a few challenges for vehicular communication, the future deployment of VANET remains unpredictable. Information dissemination, security the privacy, and Internet integration are the challenges included in it. Efficient wireless communication is the most important key factor; therefore, the employed protocols and mechanism should be robust, reliable, and scalable to numerous vehicles.

VANETs differ from traditional ad hoc networks by possessing not only rapid changes in wireless links but also different network densities. For example, vehicular networks in urban areas are more often to form more dense networks during more traffic. In other words, in less populated rural highways or during the late-night hour the vehicular networks experience more frequent network disconnections. VANET is meant to satisfy a wide range of applications which are ranging from safety to leisure. As an outcome, the routing algorithm must be efficient and capable of adjustable to vehicular network characteristics and applications. Till now, a lot of research has been taken place to focus on an analyzing routing algorithm in many dense networks with the supposition that a typical vehicular network is well-connected. The combination of penetration of vehicles and wireless communication capacity remains poor, so a VANET must be dependent on existing infrastructure supports for large-scale deployment. Though in the future to observe high penetration with lesser infrastructures VANETs are expected more, and for this reason, it is important to regard the disconnected work problem. In VANET the decisive research challenge for developing a reliable and better performing routing protocol is a network disconnection.

Thirdly, low QoS performance. To keep the best QoS for packets forwarding is very important but difficult in urban scenarios. The achievable reasons are given as follows: (1) urban environments are normally large-scale scenarios but the communication distance from a source to its destination may be very large, (2) global QoS of candidate routing paths are not so easy to be known by a source vehicle, so in largescale networks, the routing exploration processes are always based on local traffic information with random characteristics, which provides an outcome in the end-to-end routes and also contains network-partitioned/congested road segments, (3) the process of packets forwarding can be disturbed by most of the long-established VANET routing protocols lack self-adaptation features and cannot cope with topology changes availably, and (4) verity of communication pairs are normally absent of cooperation and they do not utilize the traversed routing paths, so large number of routing exploration processes are implemented, which may lead in the outcome of redundant overhead and higher transmission delay.

Fourthly, scalability and stability. The most important and necessary step toward the realization of effective vehicular communications is to guarantee a stable and scalable routing mechanism over VANETs. Therefore, the routing paths are disrupted frequently due to varying vehicle mobility and network topologies, and it is also very difficult to ensure their stability. Moreover, in large-size urban environments, the end-to-end node-based or intersection-based source-driven routing paths are not available as they cannot handle the scalability issue.

Therefore, the future work would consider developing an adaptive MAC that incorporates the proposed channel model into the adaptive MAC for better performance.

## Figures and Tables

**Table 1:** Variable Notation.

Notation	Meaning
x	Vehicle
$C_{x}$	Throughput Achieved
y	Channel
$V_{x}$	Channel set allocated to vehicle x
$l_{xy}$	Likelihood for channel y accessibility
$1-\underset{y\in V_{x}}{\prod }l_{xy}^{\prime }$	Likelihood for channel y accessibility for atmost one channel
$l_{xy}^{\prime }$	Likelihood that channel y is not accessible
$\delta C_{x}$	Throughput increment
$V_{z}$	Input set of accessible channels
$C_{x}^{z}$	Throughput before channel allocation $y_{x}^{\prime }$.
$C_{x}^{q}$	$\mathrm{Throughput}\;\mathrm{after}\;\mathrm{channel}\;\mathrm{allocation}\;y_{x}^{\prime }$.
$y_{x}^{\prime }$	channel allocation
D	MAC Overhead
T	Number of vehicles
j	Vehicle
$P_{j}$	A set of channels shared by j
$F_{y}$	Group of vehicles who share channel y
$P_{o}$	A set of channels shared by o vehicle
A	contention window
$l_{n}\left(m,\;g^{+ve}\right)$	User minimum pay cost from time to time
$g^{+ve}$	Reduction function of v
T	total number of channels in the network
Nth	time slot
xth RSU	Roadside unit x
gth RSU	Roadside unit after x
M	Data packet

**Table 2:** Evaluation Simulation Parameters.

Parameters	Network	MAC	Modulation Scheme	Mobility	Bandwidth	Frequency Channels	Vehicles	Time Slots	Environment
**Value**	10 km × 10 km	ENCCMA & DMCA	64-QAM	20 Per Frame	27 Mbps	7	30, 60 & 90	8 μs	Rural, city & highway

**Table 3:** Channel parameters [65].

Environment	City	Highway	Rural
**Path loss**	1.61	1.85	1.79
**Shadowing deviation**	3.4	3.2	3.3

**Table 4:** State-of-the-art-techniques comparison.

	DMCA (Ours)	ENCCMA	MS-ALOHA	SLOP	EDF-CSMA
Environment	C.H.R	flowing vehicles freely	highway and urban	driver intelligent	NA
Algorithm	DMCA	(NCC-FDMA-TDMA)	MS-ALOHA	Wave-Slotted aloha	EDF-CSMA
Vehicle varied Density	Yes	No	No	No	No
Simulator used	SIMITS	SIMITS	VISSIM	YES (NA)	NS-3
MAC USED	802.11p MAC	802.11p MAC	802.11p MAC	802.11p MAC	802.11p MAC
Mobility	Yes	Yes	Yes	Yes	Yes
Channel sharing available	Yes	Yes	No	No	No
Reference	(Ours)	[57]	[66]	[67]	[68]

**Table 5:** Comparison of resource allocation techniques for V2X networks.

Use Case	Objectives	Method	RSU/BS Assisted	Parameters	Scenario	Mobility	Reference
Sheared and Un-sheared nodes channels	Maximizing throughput, Minimizing collision	Distributed Medium Channel Allocation (DMCA)	yes	Bandwidth	City, Highway, Roral	Yes	Ours
Generic	Interference Minimizing	Subpool sensing-based algorithm	No	Bandwidth	Urban grid layout	Yes	[69]
Generic	Maximizing Connectivity	Graph theory	Yes	Bandwidth	Single-Lane Highway	No	[70]
Generic	Maximizing throughput	Graph theory	Yes	Bandwidth	Single-Lane Highway	Yes	[71]
Generic	Maximizing sum rate	Hungarian method	Yes	Bandwidth Power	Two-way urban roadway	Yes	[72]
Generic	Maximizing sum-rate; minimize latency	Karush-Kuhn-Tucker theory	Yes	Bandwidth Power	Urban grid layout	No	[73]
Security	Maximizing secrecy rate	Greedy algorithm	Yes	Bandwidth	Single-lane Highway	Yes	[74]
Generic	Maximizing ergodic capacity, reliability	Hungarian method	Yes	Bandwidth Power	Multi-Lane Highway	Yes	[75]
Generic	Reliability maximizing	Pre-scheduling	No	Bandwidth	Single-lane Highway	Yes	[76]
Generic	Maximizing concurrent reuses	Perron-Frobenius theory	Yes	Bandwidth	Urban grid layout; Single-lane Highway	No	[77]
Fog Computing	Maximizing utility model	Langranign algorithm	Yes	Bandwidth	Multi-RSU network	No	[78]
Basic Safety Message relaying	interference Minimizing	Exhaustive search algorithm	No	Bandwidth	Intersection	No	[79]
Security	Maximizing resource utilization	Dynamic semi-persistent method	Yes	Bandwidth	Highway	Yes	[80]
Cloud Computing	Maximizing discount value	Semi-Markov decision process	Yes	Computing resource	Urban area	No	[81]
Vehicle Platooning	Maximizing sum rate	Weight matching theory	Yes	Bandwidth	Single-lane Highway	Yes	[82]
Automated guided vehicle	QoS Maximizing	Lyapunov optimization	Yes	Bandwidth	Highway	Yes	[83]
Vehicle Platooning	Maximizing service guaranteed users	Conflict-Free SPS	Yes	Bandwidth	Highway	Yes	[84]
Platooning Vehicle	stability Maximizing	Application-adaptive algorithm	Yes	Bandwidth	Highway	Yes	[85]
multi platooning Vehicle	reallocation rate, Minimizing delay	Lyapunov optimization	Yes	Bandwidth Power	Highway	Yes	[86]

## Data Availability

Note applicable.
